# Collective motion of cells crawling on a substrate: roles of cell shape and contact inhibition

**DOI:** 10.1038/s41598-017-05321-0

**Published:** 2017-07-12

**Authors:** Simon K. Schnyder, John J. Molina, Yuki Tanaka, Ryoichi Yamamoto

**Affiliations:** 1 Department of Chemical Engineering, Kyoto University, Kyoto, 615-8510 Japan; 2 Fukui Institute for Fundamental Chemistry, Kyoto University, Kyoto, 606-8103 Japan

## Abstract

Contact inhibition plays a crucial role in cell motility, wound healing, and tumour formation. By mimicking the mechanical motion of cells crawling on a substrate, we constructed a minimal model of migrating cells that naturally gives rise to contact inhibition of locomotion (CIL). The model cell consists of two disks, a front disk (a pseudopod) and a back disk (cell body), which are connected by a finite extensible spring. Despite the simplicity of the model, the collective behaviour of the cells is highly non-trivial and depends on both the shape of the cells and whether CIL is enabled. Cells with a small front disk (i.e., a narrow pseudopod) form immobile colonies. In contrast, cells with a large front disk (e.g., a lamellipodium) exhibit coherent migration without any explicit alignment mechanism in the model. This result suggests that crawling cells often exhibit broad fronts because this helps facilitate alignment. After increasing the density, the cells develop density waves that propagate against the direction of cell migration and finally stop at higher densities.

## Introduction

Directional collective motion of cells is fundamentally important for embryogenesis, wound healing and tumour invasion^1–5^. Cells move in clusters, strands or sheets to cover empty areas^6^, grow or invade tissues. The manner in which the cells coordinate and control their motion is the subject of ongoing research. At the level of a single cell, it is well-established that a cell’s motion is intricately linked to its shape. The shape of crawling cells is highly variable and depends on the type of cell, the substrate and aspects of the migration process itself^7–10^. To move, a cell needs to break symmetry^8^, as a circular cell does not move. While there is evidence that shape has a strong influence on scattering and can lead to clustering and collective directed motion in the case of active swimming particles^11, 12^, less is known regarding the role of cell shape in the organization of collective crawling. It has been shown in simulations that inelastic collisions between crawling cells, e.g., due to deformation, can lead to coherent migration^13–17^, which suggests that deformation is important for collective cell behaviour.

When crawling cells come into contact, their protrusions are inhibited, which tends to change their shape and orientation^18, 19^. It was shown that this effect, which is called contact inhibition of locomotion (CIL), enables cells to follow chemical gradients more effectively by aligning them^20, 21^. In growing colonies, CIL leads to a slowdown of the motility of individual cells when the density of their environment crosses a certain threshold^22^. Thus, CIL is believed to play a crucial role in the control of collective tissue migration^15, 20, 23–26^, tissue growth^22, 27^, morphogenesis, wound healing and tumour development^28^. The behaviour of cells undergoing CIL depends on many factors, such as the presence of cell adhesion molecules and receptors, and different types of cells can exhibit different types of CIL^23^. This variation has made it difficult to produce a unified description of CIL behaviour^23^ but creates an opportunity to build minimal models that capture some if not the entire range of the behaviours found in experiments.

Clearly, CIL, cell shape and deformability are linked. Evidence indicates that CIL is primarily based on biochemical interactions between cells with mechanical interactions playing a secondary role^23^. However, in the minimal model presented here, we do not assume there is any particular mechanism underlying CIL but use a phenomenological model instead. From such a point of view, it is merely important to reproduce the characteristic behaviour of the cells and not the internal details; cells that come into contact with other cells are inhibited in their locomotion and slow down or change their direction in response. As we will demonstrate, a mechanical model is sufficient for this. Therefore, we built a minimal model of cells crawling on a substrate to isolate behaviour caused purely by the interplay of contact inhibition and deformable shape while neglecting properties such as cell-cell adhesion, biochemical interactions between the cells, and chemotaxis. The simplicity of our model enables us to simulate considerably larger systems than can be simulated using more complex models, and this ability minimizes the finite size effects.

The model is based on the currently accepted process of a cell crawling on a surface^29–31^. Before it begins migrating, the cell polarizes, and the front and back become distinguishable. Then, the cell extends protrusions, such as pseudopods, that are driven forward by actin polymerization. The protrusions adhere to the substrate through adhesion sites, over which the cell exerts traction forces. Adhesion sites at the back of the cell are released and pulled in as the actin cytoskeleton depolymerizes. In our model, the cells are represented by two disks, connected by a finitely extensible spring. The cell migrates by expanding the spring, with the front disk exerting a motility force on the substrate. We speculate on a mechanism of contact inhibition in which the cell motility is proportional to the extension of the cell, which was motivated by the observation that cell speed depends on the extension of the pseudopods^32^. An alternative motility term in which the force is constant was used for comparison.

From such a minimal model, quantitative agreement with experiments cannot be expected, but we found qualitative agreement with a wide range of properties of crawling cells. Consistent with observations in cell colonies, such as the formation of colonies by Madin-Darby canine kidney epithelial (MDCK) cells^33^, we found that the average cell speed decreases strongly with cell density, which is an effect that vanishes when contact inhibition is switched off. The cell speed distributions are similar to those of fibroblasts^34^. Furthermore, we found a dynamic phase transition that depends on cell shape. When the front is larger than the back, which is typical of many migrating cells (e.g., keratocytes and fibroblasts)^34^, the cells exhibit coherent migration, even though there is no explicit alignment mechanism included in the model. This result suggests that the broad front often observed in crawling cells helps them achieve coherent motion. When contact inhibition is switched off, we found weakened alignment. The transition from disorder to order that occurs with keratocytes when their density is increased^35^ arises in the model when cell noise is included. Finally, before arresting at high density, the system exhibits strong density and velocity fluctuations in which dense regions of arrest travel against the average direction of motion. This phenomenon is also seen as traffic jams that spontaneously arise in traffic flow. Similar waves have been recently observed^36^, and our results could link these observations to the contact inhibition of locomotion.

## A simple model of crawling cells

**Figure 1:**
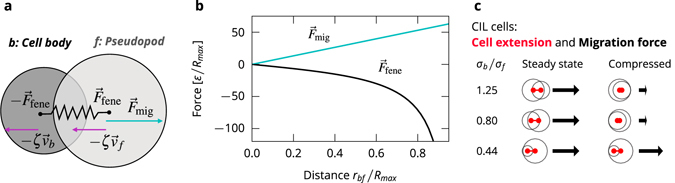
Illustration of the cell model. (**a**) Schematic of a cell ﻿w﻿ith a finite extensible non-linear elastic spring connecting the disks and exerting the force ${\overrightarrow{F}}_{fene}$ (Eq. 1), the migration force ${\overrightarrow{F}}_{mig}$ acting on the front disk (Eq. 2), and the friction forces -ζ${\overrightarrow{v}}_{b}$ and -ζ${\overrightarrow{v}}_{f}$. (**b**) Forces acting on the two disks composing a cell separated by a distance ${r}_{bf}=|{\overrightarrow{r}}_{bf}|$ (Eq. 1, 2). (**c**) Cell extension *r*_*bf*_ (red line segment) and migration force (black arrow) for the CIL cells in the steady state and a compressed state in which the disks are fully overlapping.

Each cell consists of a cell body and a pseudopod, which are modelled as disks with diameters of *σ*_*b*_ and *σ*_*f*_ at the positions ${\mathop{r}\limits^{\longrightarrow}}_{b,f}$, where ${\mathop{r}\limits^{\longrightarrow}}_{bf}={\mathop{r}\limits^{\longrightarrow}}_{f}-{\mathop{r}\limits^{\longrightarrow}}_{b}$ is the separation of the two disks (Fig. 1a). To allow for different cell shapes, *σ*_*b*_ and *σ*_*f*_ can be different. The disks experience a drag force with the substrate $-{\zeta }_{i}{\mathop{v}\limits^{\longrightarrow}}_{i}$, where *ζ*_*i*_ is the friction coefficient and ${\mathop{v}\limits^{\longrightarrow}}_{i}$ is the velocity of the disk *i* ∈ *f*, *b*. Assuming that the substrate friction is large compared with both cell-cell and intracellular friction, we neglect the latter two friction sources and set *ζ* = *ζ*_*b*_ = *ζ*_*f*_ for simplicity. The two disks are connected by a finite extensible non-linear elastic (FENE) spring^37^ representing the cytoskeleton

1 $${\mathop{F}\limits^{\longrightarrow}}_{{\rm{fene}}}({\mathop{r}\limits^{\longrightarrow}}_{bf})=-\frac{\kappa {\mathop{r}\limits^{\longrightarrow}}_{bf}}{1-{({r}_{bf}/{R}_{{\rm{\max }}})}^{2}},$$

where *κ* is the spring constant (Fig. 1b). The characteristic length scale is set by the maximum separation of the disks due to the FENE spring, *R*_max_.

The migration force *F*_mig_ is applied *to the front disk* as a linear function of the separation of the two disks ${\mathop{r}\limits^{\longrightarrow}}_{bf}$

2 $${\mathop{F}\limits^{\longrightarrow}}_{{\rm{mig}}}({\mathop{r}\limits^{\longrightarrow}}_{bf})=m{\mathop{r}\limits^{\longrightarrow}}_{bf}$$

with *m* as an adjustable parameter (Fig. 1b). The cell is only motile when its disks have non-zero separation, *r*_*bf*_ > 0, thus, when its shape deviates from a circle. Such coupling of motility and deformation is typical in crawling cells^38^ and forms the basis for CIL in our model.

For comparison, we used a version of the model without CIL. We replaced the linear migration force term with a force of constant strength such that it leaves ${r}_{bf}^{{\rm{ss}}}$ and *v* ^ss^ unchanged

3 $${F}_{mig}=m{r}_{bf}^{{\rm{s}}{\rm{s}}}{\hat{r}}_{bf}(\text{no}\mbox{-}\text{CIL}\,\text{version}),$$

where ${\hat{r}}_{bf}={\mathop{r}\limits^{\longrightarrow}}_{bf}/|{\mathop{r}\limits^{\longrightarrow}}_{bf}|$ is the unit vector providing the cell orientation. In this model version, no-CIL cells will always exert exactly the same migration force, regardless of whether the local environment allows for extension of the cell. This makes the system more similar to Vicsek models with a constant speed^39–41^. Note that Stramer and Mayor^23^ use the term ‘uninhibited cells’ for cells that are able to crawl over each other when colliding. We do not consider this case and instead refer to cells with a constant migration force as uninhibited. In the nomenclature of Stramer and Mayor^23^, our uninhibited cells simply represent a different type of CIL.

Disks belonging to different cells interact via the short-range, purely repulsive Weeks-Chandler-Andersen potential^42^ because interactions occur mainly through direct contact. For the interaction of a pair of disks *α* and *β* (*α*, *β* ∈ [*b*, *f*]) belonging to different cells at a separation of ${\mathop{r}\limits^{\longrightarrow}}_{\alpha \beta }={\mathop{r}\limits^{\longrightarrow}}_{\beta }-{\mathop{r}\limits^{\longrightarrow}}_{\alpha }$, the force on disk *α* is given by

4 $${\mathop{F}\limits^{\longrightarrow}}_{{\rm{WCA}}}({r}_{\alpha \beta })=\{\begin{array}{ll}-24\varepsilon [2{(\frac{{\sigma }_{\alpha \beta }}{{r}_{\alpha \beta }})}^{12}-{(\frac{{\sigma }_{\alpha \beta }}{{r}_{\alpha \beta }})}^{6}]{\mathop{r}\limits^{\longrightarrow}}_{\alpha \beta }/{r}_{\alpha \beta }^{2}, & {r}_{\alpha \beta } < {r}_{{\rm{cut}}},\\ \mathrm{0,} & r\ge {r}_{{\rm{cut}}}.\end{array}$$

where the interaction diameter is given by *σ*_*αβ*_ = (*σ*_*α*_ + *σ*_*β*_)/2 and the energy scale is given by *ε*. The force acting on disk *β* has the same magnitude but the opposite sign. The cut-off at *r*_cut_ = 2^1/6^ *σ*_*αβ*_ makes the inter-cellular forces in the present model purely repulsive, but the inclusion of attractive terms modelling inter-cellular adhesion would be straightforward. The model has no random component, which is a valid assumption when the dynamics are dominated by collisions^43, 44^, as is typical at intermediate and high cell densities.

For each of the cells, we now have two coupled equations of motion that assume over-damped dynamics,

5 $$\begin{array}{rcl}\frac{{\rm{d}}}{{\rm{d}}t}{\mathop{r}\limits^{\longrightarrow}}_{b} & = & \frac{1}{\zeta }(-\,{\mathop{F}\limits^{\longrightarrow}}_{{\rm{fene}}}({\mathop{r}\limits^{\longrightarrow}}_{bf})+\sum _{{\rm{neigh}}.}{\mathop{F}\limits^{\longrightarrow}}_{{\rm{WCA}}})\\ \frac{{\rm{d}}}{{\rm{d}}t}{\mathop{r}\limits^{\longrightarrow}}_{f} & = & \frac{1}{\zeta }({\mathop{F}\limits^{\longrightarrow}}_{{\rm{fene}}}({\mathop{r}\limits^{\longrightarrow}}_{bf})+{\mathop{F}\limits^{\longrightarrow}}_{{\rm{mig}}}({\mathop{r}\limits^{\longrightarrow}}_{bf})+\sum _{{\rm{neigh}}.}{\mathop{F}\limits^{\longrightarrow}}_{{\rm{WCA}}}).\end{array}$$

We chose $\kappa =2.00\cdot {10}^{4}\varepsilon /{R}_{{\rm{\max }}}^{2}$ and $m=4.14\cdot {10}^{4}\varepsilon /{R}_{{\rm{\max }}}^{2}$, such that *m* = 2.07*κ*. For a derivation of this model from a more complex description, which more closely mimics the periodic crawling cycle of cells, we refer the reader to the Methods section.

If cells are undisturbed by collisions, they will eventually enter a steady state in which the forces acting on the cell balance.

$$0=\frac{{\rm{d}}}{{\rm{d}}t}{r}_{bf}=\frac{1}{\zeta }(m{r}_{bf}^{{\rm{ss}}}-\frac{2\kappa {r}_{bf}^{{\rm{ss}}}}{1-{({r}_{bf}^{{\rm{ss}}}/{R}_{{\rm{\max }}})}^{2}}).$$

In this steady state, the extension $|{\mathop{r}\limits^{\longrightarrow}}_{bf}|={r}_{bf}^{{\rm{ss}}}$ and crawling speed *v* ^ss^ of the cells are constant.

6 $$\begin{array}{l}{r}_{bf}^{{\rm{ss}}}={R}_{{\rm{\max }}}\sqrt{1-2\kappa /m},\,\,{v}^{{\rm{ss}}}={r}_{bf}^{{\rm{ss}}}m\mathrm{/(2}\zeta \mathrm{)}.\end{array}$$

Because the length of the cells is of the order *R*_max_ (see Table 1 in the Methods), the characteristic time scale of migration is given by *τ*_mig_ = *R*_max_/*v* ^ss^ = 2.57 · 10^−4^ *ζ* *R* ^2^_max_/*ε*. This is the amount of time it takes for a solitary cell in the steady state to travel approximately its own length.

**Table 1:** Size parameters for the cells. Cell length is given as the length of the cell in its steady state $({\sigma }_{b}+{\sigma }_{f})/2+{r}_{bf}^{{\rm{ss}}}$.

*σ*_*b*_/*σ*_*f*_	*σ*_*b*_	*σ*_*f*_	Cell length
1.25	0.55 *R*_max_	0.44 *R*_max_	0.68 *R*_max_
0.80	0.44 *R*_max_	0.55 *R*_max_	0.68 *R*_max_
0.44	0.27 *R*_max_	0.60 *R*_max_	0.62 *R*_max_

A side effect of coupling the migration force to the cell extension ${\overrightarrow{r}}_{bf}$ is that the migration force of a cell in a compressed state depends on the cell shape, which is defined by its diameters *σ*_*b*_ and *σ*_*f*_ (see Fig. 1c). For cells with an aspect ratio *σ*_*b*_/*σ*_*f*_ close to 1, compressing the cell brings the disk centres close to each other and significantly lowers the migration force, while a cell with a more extreme aspect ratio retains a higher migration force under a similar degree of compression.

## Collective dynamics

Cells are placed in random positions in a square simulation box of length *L* with periodic boundary conditions at area fraction *φ* = *AN*/*L* ^2^, with *N* indicating the total number of cells and *A* representing the area of a single cell in its steady state. Configurations at large *φ* were obtained by starting from systems at a lower area fraction and randomly letting individual cells divide into two new cells if there was enough free space. To minimize finite size effects, we simulated systems with up to 10^5^ cells. We integrated the equations of motion until the steady state was reached. All results are averaged over 10 independent runs.

**Figure 2:**
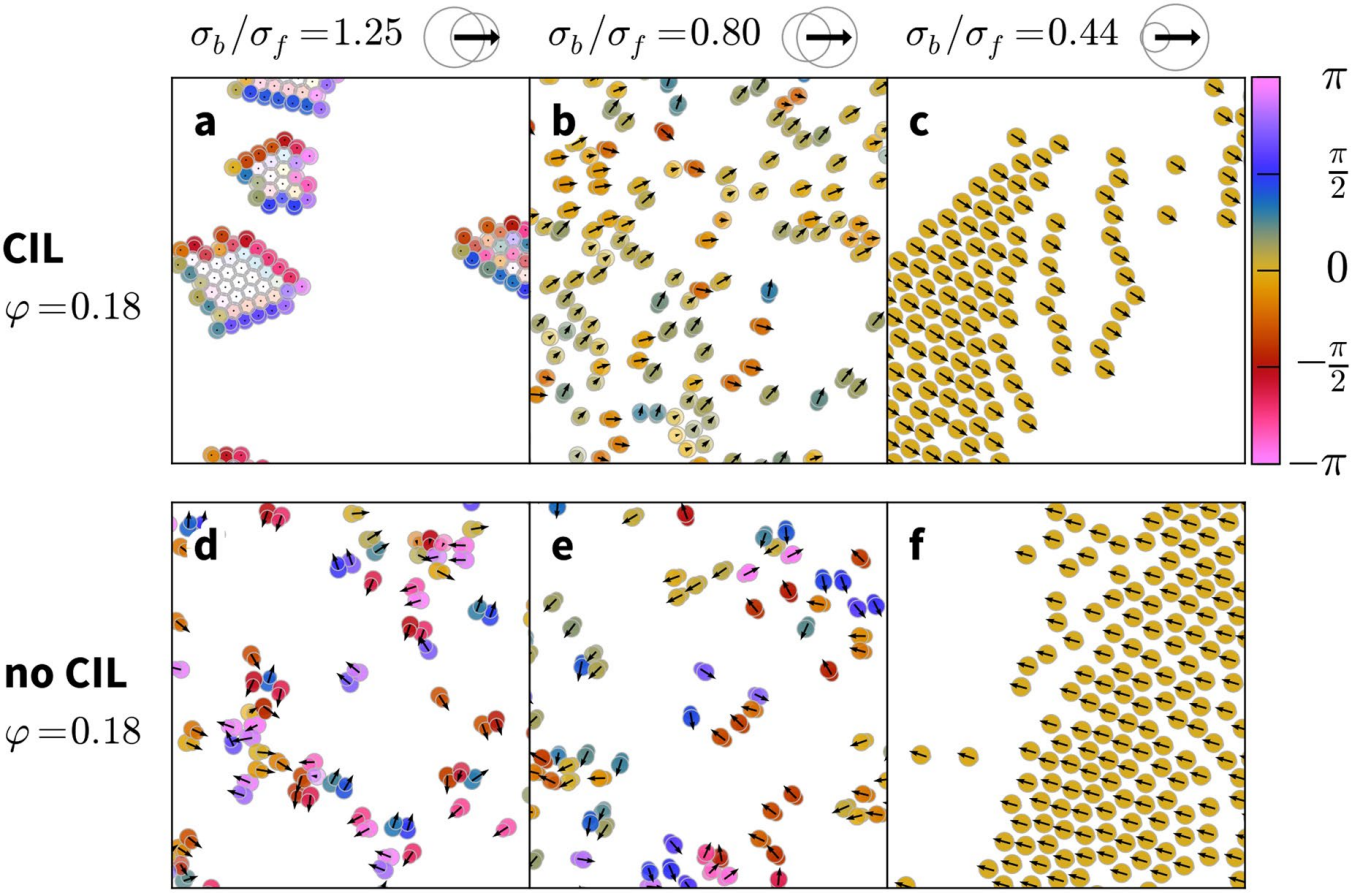
Dynamic states. Snapshots of CIL and no-CIL cells for a range of cell shapes. Cell velocities are presented as arrows, and cell extensions are displayed as colour. Hue indicates deviation from the average direction, and compressed cells are lighter in colour. (For videos, rendered with Ovito﻿^61^, see the Supplemental materials).

We first present a short overview of the dynamic states exhibited by our model. CIL cells form mostly immobile colonies when the back disk is larger than the front, *σ*_*b*_/*σ*_*f*_ > 1, see Fig. 2a). When the front disk is larger than the back disk, *σ*_*b*_/*σ*_*f*_ < 1, the CIL cells exhibit coherent migration (Fig. 2b). If the front is much larger than the back, the cells completely align and form dense, travelling bands (Fig. 2c). Uninhibited cells do not form colonies and exhibit weaker alignment at *σ*_*b*_/*σ*_*f*_ = 0.80. We will now discuss the collective cell dynamics in detail, compare our results with experimental data, and then present results of an analysis of binary collisions to explain how the observed dynamics arise.

### Cell speeds

**Figure 3:**
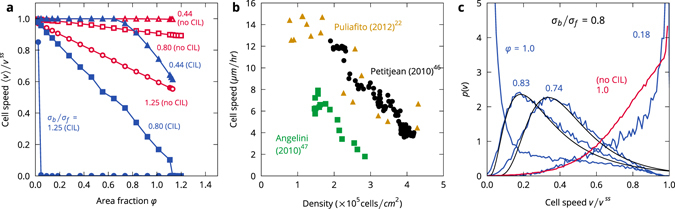
Cell speeds. (**a**) Average cell speed, normalized by the steady-state speed of a solitary cell *v* ^ss^, with (closed symbols) and without (open symbols) contact inhibition for a range of cell shapes. (**b**) Speed of MDCK cells in a growing cell colony (extracted from ref. 22) and in confluent monolayers (extracted from refs 46, 47). (**c**) Speed histograms at *σ*_*b*_/*σ*_*f*_ = 0.80. Black lines are fit to the data with a log-normal distribution.

Migrating cells slow down significantly at high cell densities^22, 45–47^. To test this effect in the model, we measured the average cell speed while varying the area fraction of the cells at three aspect ratios *σ*_*b*_/*σ*_*f*_ = 1.25, 0.80 (the inverse case), and 0.44 (Fig. 3). For *σ*_*b*_/*σ*_*f*_ = 1.25, the cell speed vanishes for all but the smallest cell density due to the formation of jammed clusters. In the opposite case, *σ*_*b*_/*σ*_*f*_ = 0.80, the contact-inhibited cells crawl at maximum speed only at very low densities. The speed decreases linearly over the entire density range as the cells become increasingly more inhibited by collisions with neighbouring cells. The cells fully arrest when they are close-packed at *φ* ≈ 1.1 (area fractions can be larger than 1 because the disks are soft and because the area fraction is defined based on the cell’s largest possible area, which it attains in its steady state). At *σ*_*b*_/*σ*_*f*_ = 0.44, the cells crawl at maximum speed up to *φ* ≈ 0.6, where a slowdown occurs. The higher speeds are enabled by their weaker contact inhibition response (Fig. 1c). In comparison, the uninhibited cells at corresponding aspect ratios show a much weaker response to increasing density. The strong slowdown of the CIL cells with big fronts is comparable to the behaviour of MDCK cells (Fig. 3b). Even though MDCK cells are adhesive with a wide variability in cell area while our cells are not adhesive and vary minimally in their area, our model’s coupling of cell extension and motility already gives rise to a known relationship between cell density and speed. Note that Garcia *et al*.^48^ recently suggested that the slowdown in MDCK cells may not be primarily driven by density but by cell-cell and cell-substrate adhesion. This effect is not captured in our model.

The distribution of cell speeds in the aligning case, e.g., *σ*_*b*_/*σ*_*f*_ = 0.80 (Fig. 3c), depends strongly on density as well. At low densities, most cells move at maximum speed *v* ^*ss*^, while most cells are arrested at high densities. At intermediate densities, a distribution with a non-Gaussian tail arises. Such distributions are found in fibroblasts in monolayers^32, 49, 50^. In this scenario, this distribution only arises in the CIL cells, which points to the relevance of CIL in establishing typical collective crawling cell dynamics. A key difference between the model and experiments, as reported by Vedel *et al*.^32^, is that the speed distribution does not depend on density (including non-confluent and confluent systems), which is impossible in this scenario because of the slowdown effect of our CIL mechanism.

### Collective alignment

**Figure 4:**
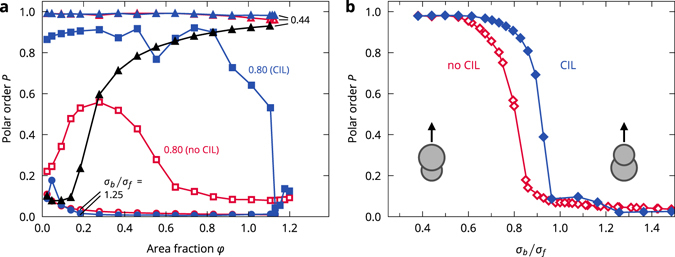
Collective alignment. (**a**) Polar order *p* as a function of area fraction for a range of cell shapes. Simulations with cell noise at *σ*_*b*_/*σ*_*f*_ = 0.44 are shown in black. (**b**) Polar order *p* as a function of cell shape at area fraction *φ* ≈ 0.18.

The coherent motion of cells can be measured with the polar order *P*, which is an order parameter of collective alignment

7 $$P=|\langle {\mathop{r}\limits^{\longrightarrow}}_{bf}/|{\mathop{r}\limits^{\longrightarrow}}_{bf}|\rangle |,$$

which is 1 for full alignment of the cells and 0 for fully random or isotropic orientations (Fig. 4). When the CIL cells form clusters, e.g., at *σ*_*b*_/*σ*_*f*_ = 1.25, the order parameter vanishes as the orientations of the cells mostly point toward their cluster’s centre. This leads to a full arrest of the cells. At *σ*_*b*_/*σ*_*f*_ = 0.80, the cells are well-aligned at most densities. Alignment weakens as the cells approach full arrest. At *σ*_*b*_/*σ*_*f*_ = 0.44, the cells are completely aligned at all densities. At densities where it is possible for the cells to be spaced far enough from each other so they do not interact (at approximately *φ* ≤ 0.65), there are few to no collisions and the cells crawl at full speed. For *σ*_*b*_/*σ*_*f*_ = 0.80, where alignment is not perfect, frequent collisions lower the average speed of the coherently moving cells. The non-CIL cells at *σ*_*b*_/*σ*_*f*_ = 1.25 do not form clusters and move in a disordered manner with a vanishing polar order. At *σ*_*b*_/*σ*_*f*_ = 0.80, the cells show some alignment, especially at intermediate densities, but are always more weakly aligned than the corresponding CIL cells. At *σ*_*b*_/*σ*_*f*_ = 0.44, the cells achieve near-perfect orientational order at all densities, just as the CIL cells do. By performing additional simulations for a wide range of shapes (Fig. 4b), we found that collective alignment quickly arises for the CIL cells at a critical shape ratio of *σ*_*b*_/*σ*_*f*_ ≈ 0.95. The transition for no-CIL cells occurs at lower aspect ratios, *σ*_*b*_/*σ*_*f*_ ≈ 0.8, but is qualitatively similar. Therefore, the alignment transition is shifted according to the CIL mechanism but is not fundamentally changed.

## Binary collision analysis

**Figure 5:**
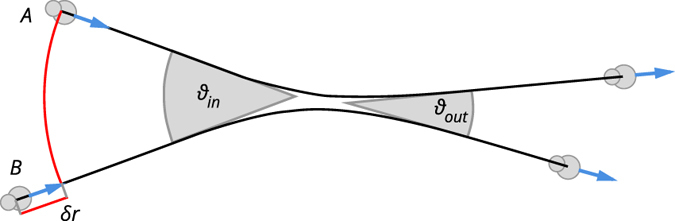
Binary collision. Schematic view of a collision of two cells, labelled A and B, with an incoming angle *ϑ*_*in*_ and outgoing angle *ϑ*_*out*_. The difference in the distances of the cells to the collision centre before the collision is given by *δr*.

To provide a framework for understanding how the collective dynamics are derived from the shape and CIL mechanism, we analysed collisions between pairs of cells (Fig. 5). We parameterized the collisions with two parameters, the incoming angle *ϑ*_*in*_ and the offset distance *δr*. The directions of motion of the cells far from the collision are defined as *ϑ*_*in*_. We define the collision centre as the point at which the paths of the cells would cross if there were no interaction between them. A symmetric collision occurs when both cells are at the same distance from the collision centre. To investigate asymmetric collisions, we increased the distance of one of the cells to the collision centre by an offset *δr*. Finally, we characterized the outcome of a collision with the outgoing angle *ϑ*_*out*_ between the directions of cell motion after the collision. Collision alignment can be estimated by the difference *δϑ* between the outgoing and incoming angles,

8 $$\delta \vartheta ={\vartheta }_{out}-{\vartheta }_{in}.$$

Aligning collisions correspond to *δϑ* < 0, and disaligning collisions correspond to *δϑ* < 0.

### Head-on collisions

**Figure 6:**
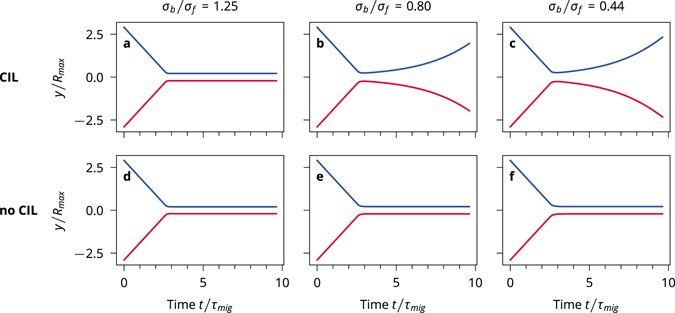
Head-on collisions. Y-coordinates for two cells of the same type colliding with a collision angle of *θ*_*in*_ = *π*, for all the different cell types.

The simplest scenario is two cells colliding *exactly* head on, *ϑ*_*in*_ = *π* (Fig. 6). CIL cells with a small front, *σ*_*b*_/*σ*_*f*_ = 1.25, collide, become compressed by the collision and come to rest. Due to the compression, their migration forces are lowered. This tendency allows for the formation of clusters. However, we found by inspecting videos of our simulations that cluster formation typically requires the collision of multiple cells with each other in close succession, see the Supplemental materials. CIL cells with a large front, *σ*_*b*_/*σ*_*f*_ = 0.80 and 0.44, collide and then reverse their motion. This is a typical response of CIL cells^23, 51^. The reversal of the motion occurs in our model because for most of the duration of the collision, the front disks are repelling each other strongly, while the back disks do not register the presence of the other cell. This effect compresses the cell and lowers the migration force. Finally, when the cell is completely compressed, the centre of the front disk is pushed over the centre of the back disk and the cell reverses its direction.

The uninhibited cells all come to a stand-still in a head-on collision with the migration forces of both cells completely cancelling each other out. Because compressing a no-CIL cell does not lower the migration force, the cells resist reversal of their crawling direction. This is not necessarily an unrealistic result, as it represents a possible contact inhibition response^23^.

### Polar order due to cell shape

**Figure 7:**
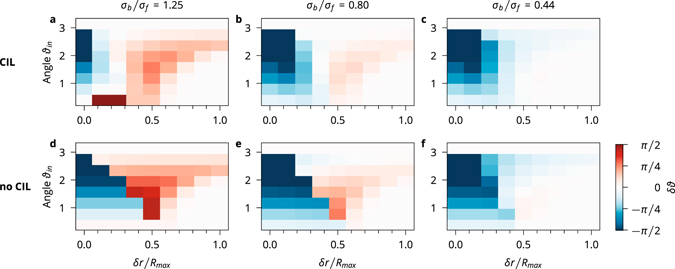
Polar order due to cell shape. Difference *δϑ* between outgoing and incoming angles as a function of the incoming angle *ϑ*_*in*_ and *δr*, which is the difference in the distances of the cells from the collision centre before the collision.

**Figure 8:**
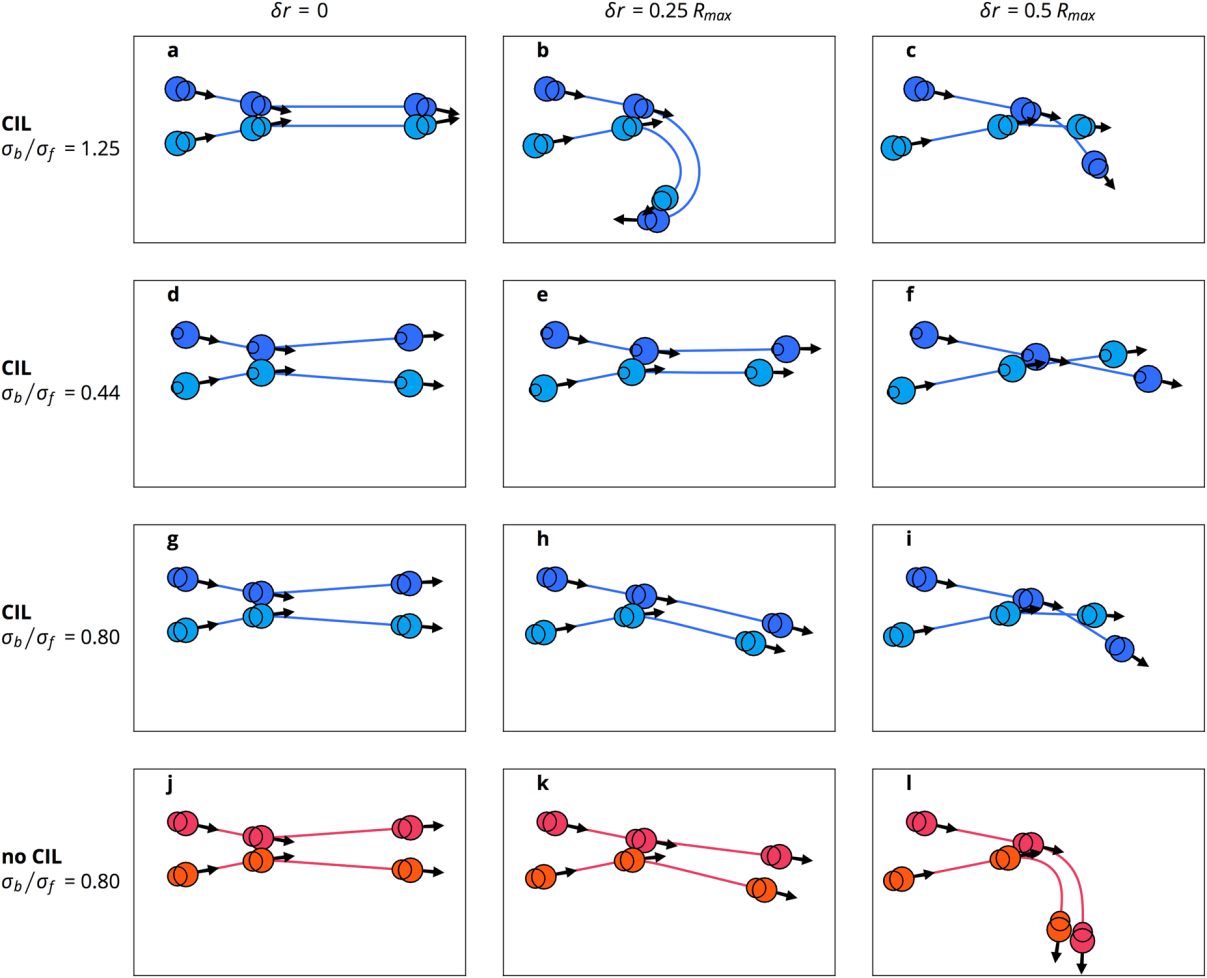
Binary collisions. Trajectories of two cells colliding with a collision angle of *θ*_*in*_ = *π*/8. Arrows indicate the migration forces. The cells are shown at their initial position, during the collision, and at the end of the simulation. All trajectories depict the same duration of time.

Evaluating *δϑ* for all collisions reveals that collisions between cells with small fronts are mostly disaligning, while collisions between cells with large fronts are mostly aligning (Fig. 7). This effect can be illustrated with exemplary trajectories (Fig. 8) in which the same collisions are shown for cells with different shapes. The most instructive scenarios are collisions with a small incoming angle. They are representative of a system that is mostly ordered and allow for easy verification of whether the order is stable.

When cells with small fronts collide symmetrically or with a small *δr*, see panel a), they often end up paired with the migration forces pushing them together. For larger *δr*, the cell in the back tends to run into the back of the front cell (because the back is big), see panel b, c). This collision exerts a torque on the front cell, which pushes it into the path of the back cell. This effect either deflects the back cell strongly as well, see panel b), or increases the angle between the two cells, see panel c). This effect can be strong enough to completely reverse the direction of motion of the cells and completely disrupt the polar order.

When two cells with big fronts collide symmetrically, the collision is aligning, see panel d). Because the front disks of the cells are bigger than the back disks, the cells mostly interact through contact of their respective fronts. The collision gives the cells a torque that turns them away from each other. For asymmetric collisions the results are very similar, see panel e). In panel f), *δr* is so large that the cells only brush each other in passing, which leaves the angle between them nearly unchanged.

In summary, if a cell coming from behind hits the front disk of the front cell, it tends to align the cells, while hitting the back disk tends to disalign them. Cells with big fronts have a higher probability of being hit in the front than cells with a small front, which is why cells with big fronts exhibit polar order and cells with small fronts do not.

The manner in which CIL helps cells to align can be understood intuitively by analysing a few characteristic collisions. While symmetric collisions are very similar between CIL and no-CIL cells, see panels g, j), differences are more pronounced in asymmetric collisions. When two CIL cells collide, the cell in the back tends to become more compressed, slows down more and lets the cell in front move away, see panels h, i). This type of collision reduces the time during which the cells interact with each other and leads to weaker deflections than those associated with no-CIL cells, see panels k, l). The effect is most pronounced in cases of larger asymmetry when no-CIL cells can get paired up and keep pushing each other until they strongly deviate from the original direction, see panel l). Therefore, the reason for the increased polar order in the simulations with CIL cells is their inclination to slow down and weaken the effects of the collisions.

### Scattering of cells by collision

**Figure 9:**
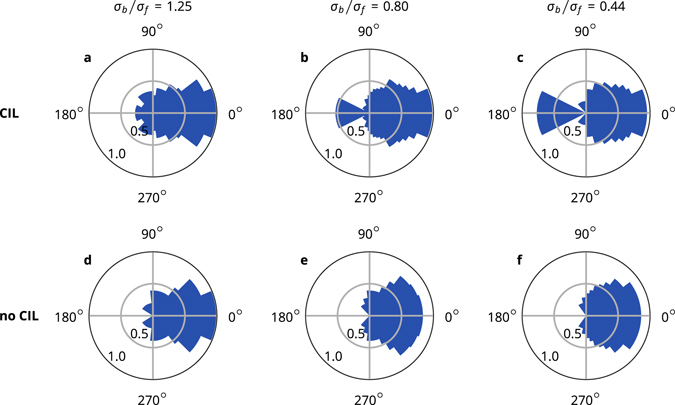
Contact inhibition increases the frequency of back-scattering. Distribution of direction of motion of a cell after a collision with another cell with 0 indicating no change in the direction.

From the distribution of scattering angles after the collision, which is defined as the angle between the incoming direction of motion and the outgoing direction of motion of the cell (Fig. 9), we see that CIL cells have a higher probability of being backscattered than their uninhibited counterparts. Backscattering typically occurs when the collisions are nearly head-on. Note that the probability of a complete reversal of the direction of motion is not as high as that reported for *in vivo* cells, e.g., in the study of neural crest cells by Carmona-Fontaine *et al*.^19^. Nevertheless, the difference between CIL and no-CIL cells is qualitatively captured in our model.

## Velocity waves

**Figure 10:**
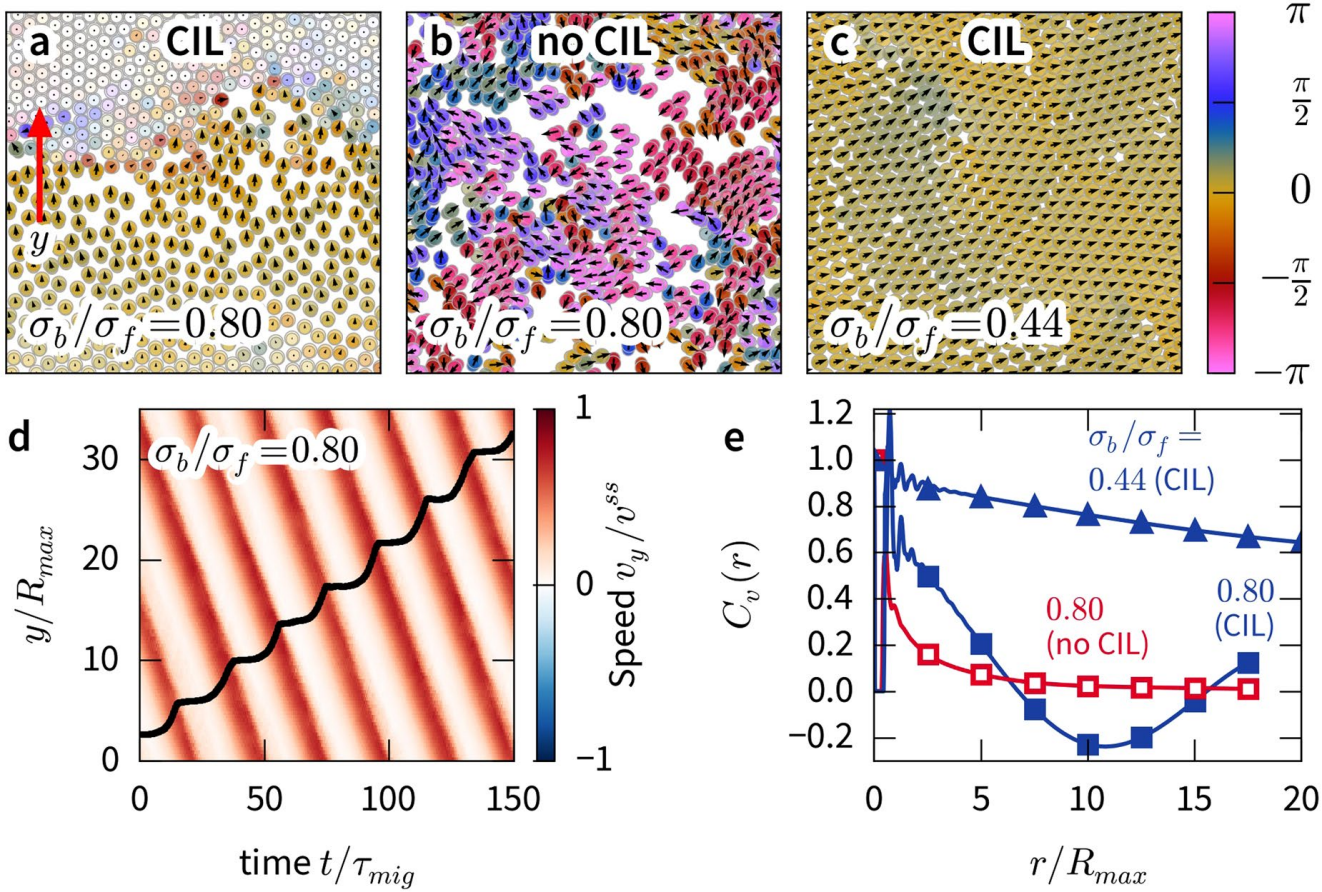
Velocity waves. (**a**–**c**) Simulation snapshots of CIL and no-CIL cells at area fraction *ϕ* = 0.92 with cell velocities shown as arrows. Hue indicates the deviation from the average direction, and slower cells are lighter in colour. (For videos, see Supplemental materials) (**d**) Kymograph of simulation shown in. (**a**) Average of cell velocities in the y-direction, see arrow in (**a**), as a function of time. Trajectory of one cell superimposed in black. (**e**) Velocity correlation functions for simulations shown in (**a**–**c**).

In the transition toward the arrest of cells with *σ*_*b*_/*σ*_*f*_ = 0.80, a remarkable feature develops, namely, areas of dynamic arrest continuously form and dissolve. The arrested areas grow in size with density until they form system-spanning waves (Fig. 10a), which suggests that there is a growing length scale. The waves travel against the direction of motion of the cells (Fig. 10d), akin to traffic jams in models of car traffic^52–54^. In this state, the cell speed distribution shows a peak at or near 0 and a long tail (see data for *φ* = 1.0 in Fig. 3c). The onset of system-spanning arrest waves roughly coincides with the decrease in the order parameter at *φ* ≈ 0.85. We do not observe such waves in the non-CIL systems (Fig. 10b). Thus, the waves are directly associated with the CIL. Furthermore, the waves only occur when contact inhibition is strong; the cells with *σ*_*b*_/*σ*_*f*_ = 0.44, which have a weaker slowdown at high densities (Fig. 1c), always travel coherently (Fig. 10c). Because they slow down less abruptly, they never fully jam.

The correlation function of cell speed fluctuations (VACF)

9 $${C}_{v}(r)=\langle {\rm{\Delta }}\mathop{v}\limits^{\longrightarrow}\mathrm{(0)}\cdot {\rm{\Delta }}\mathop{v}\limits^{\longrightarrow}(r)\rangle /\langle {\rm{\Delta }}\mathop{v}\limits^{\longrightarrow}{\mathrm{(0)}}^{2}\rangle ,$$

with ${\rm{\Delta }}\mathop{v}\limits^{\longrightarrow}(r)=\mathop{v}\limits^{\longrightarrow}(r)-\langle \mathop{v}\limits^{\longrightarrow}\rangle$ provides deeper insight into the dynamics of the systems (Fig. 10e). The VACF of the CIL cells becomes negative on the length scale of the extent of the traffic jam with a minimum at approximately 10*R*_max_. The VACF of corresponding no-CIL cells decays on the length scale of a few *R*_max_ and never becomes negative. This result reveals that cells move in small correlated clusters. Inspection of cell trajectories reveals that these clusters have short lifetimes. The highly ordered state of the CIL cells at *σ*_*b*_/*σ*_*f*_ = 0.44 corresponds to a slow decay of the VACF.

## Discussion

CIL cells with small fronts cluster because their migration force tends to stay pointed toward the other cells after a collision, which compresses and inhibits them. Similar jamming is also observed in active particle systems^55–57^. Although the jamming of cells purely due to their shape has not yet been observed, it has been shown that crawling cells may form tissue-like clusters when placed on soft substrates but scatter apart on stiff substrates^58^. Cells on stiff substrates are able to exert stronger traction forces than those on soft substrates^59^. Thus, even though we did not model substrate stiffness, the result is that our CIL cells cluster while uninhibited cells (which on average exert stronger traction forces) do not, which shows rough qualitative agreement with the findings of Guo *et al*.^58^. A caveat is that in real cell clusters, the cells tend to exhibit protrusions pointing away from the cluster, not toward it. In our model, such a situation does not lead to a stable cluster because we neglect adhesion.

As seen in Fig. 4, both CIL and no-CIL cells undergo a transition from disorder to coherent migration, which is driven by the shape asymmetry of the cells. Thus, unlike most models, the alignment mechanism is not explicitly included in the model. While such a transition as a function of cell shape has not been observed in experiments, it may explain why crawling cells often exhibit a broad front: *It improves alignment*. We would like to note, however, that the cell shape in our model does not necessarily represent the actual shape of cells, but rather, the repulsion upon collision with other cells. In particular, cells with big fronts represent the case in which cells are strongly repelled from other cells when colliding with them. Ultimately, a big front disk in our model is an additional form of contact inhibition. In this interpretation, the disorder-order state transition due to shape is ultimately a transition due to contact inhibition. The contact inhibition mechanism of the migration force in our model further enhances the alignment of crawling cells. The alignment of cells due to CIL in our model agrees with the results for neural crest cells^19, 21^ and other numerical simulations^15, 26^.

A similar transition due to cell shape occurs in the self-assembly of roughly triangular, stiff, active particles in simulations performed by Wensink *et al*.^12^. However, we found that the transition was *reversed*, namely, cells with a large front travel coherently in our model and cluster in ref. 12, while cells with a small front form clusters in our model and travel coherently in ref. 12. The most notable difference between the models and the likely reason underlying the discrepant results is that our cells are highly deformable and easily compressed during collisions, which significantly changes the collision dynamics. Indeed, deformability has been shown to lead to alignment in other numerical studies of deformable particles^14, 15, 17^. In addition, Wensink’s particles differ from our cells in that the migration force acts on the particle’s centre of mass, whereas it acts on the front disk in our model.

**Figure 11:**
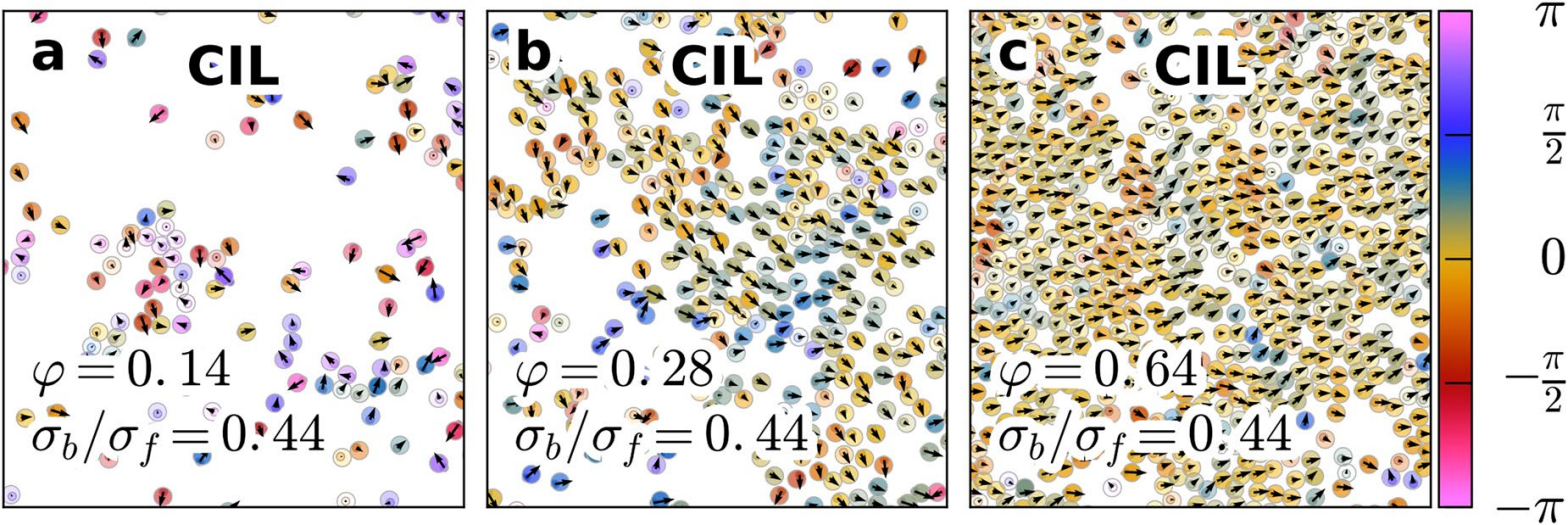
Disorder to order transition. Simulation snapshots of CIL cells with cell noise in steady states at different area fractions. The cell velocities are shown as arrows, and the colour hue indicates deviation from the average directions, with compressed cells indicated by a lighter colour. Cells randomly migrate at a low density (**a**), some coherency appears at an intermediate density (**b**), and highly coherent migration is observed at a high density (**c**). Videos for these systems are available in the Supplemental materials, which compare well with similar experimental videos for keratocytes^35^.

Keratocytes exhibit a different transition from disordered to coherent motion that is driven by an increase in density^35^. At first, this behaviour is not found in our model because the alignment of cells is mostly independent of density. However, by including a random force acting on the cell disks (see the Methods section for details), this transition occurs in the model as well (see the black line with triangles in Figs 4(a) and 11).

Backward-travelling waves have also been observed in expanding monolayer sheets of MDCK cells^36^; however, whether waves with full arrest can occur in crawling cells, e.g., in ring geometries^45, 60^, remains an open question. Because our CIL mechanism links the velocity waves to corresponding density waves, it makes them distinct from heterogeneous velocity fields occurring without corresponding heterogeneous density^47^.

## Summary and conclusion

To reveal the universal dynamics of contact-inhibited, deformable cells, we modelled crawling cells on a substrate using a minimal mechanical model. We assumed the migration force to be proportional to the extension of the cell, which naturally gave rise to contact inhibition of locomotion. We found rich dynamic behaviour in qualitative agreement with a variety of experiments, with the presence of multiple state transitions found to be a function of cell shape, cell density and whether locomotion is inhibited. Our results suggest that crawling cells may often exhibit a broad front because the broad front enhances alignment. Finally, we found density waves that propagate against the direction of cell motion. This model is a natural candidate with which to further investigate the dynamics of cellular tissues. Of particular interest are the effects of contact inhibition and cell shape on tissue growth and wound closure, as well as the dynamics of malignant cells in mixtures of contact-inhibited and -uninhibited cells.

## Methods

### Cell sizes

The area *A* of a cell with a back disk of diameter *σ*_*b*_, a front disk of diameter *σ*_*f*_ and a distance *r*_*bf*_ between the particles is given by

10 $$\begin{array}{rcl}A & = & {A}_{1}+{A}_{2}-{\rm{overlap}}\\  & = & \frac{\pi }{4}({\sigma }_{b}^{2}+{\sigma }_{f}^{2})-\frac{{\sigma }_{b}^{2}}{4}\cdot {\cos }^{-1}(\frac{4{r}_{bf}^{2}+{\sigma }_{b}^{2}-{\sigma }_{f}^{2}}{8{r}_{bf}{\sigma }_{b}})-\frac{{\sigma }_{f}^{2}}{4}\cdot {\cos }^{-1}(\frac{4{r}_{bf}^{2}-{\sigma }_{b}^{2}+{\sigma }_{f}^{2}}{8{r}_{bf}{\sigma }_{f}})\\  &  & +\frac{1}{8}\sqrt{(-2{r}_{bf}+{\sigma }_{b}+{\sigma }_{f}\mathrm{)(2}{r}_{bf}+{\sigma }_{b}+{\sigma }_{f})}\cdot \sqrt{\mathrm{(2}{r}_{bf}+{\sigma }_{b}-{\sigma }_{f}\mathrm{)(2}{r}_{bf}-{\sigma }_{b}+{\sigma }_{f})}\end{array}$$

We fix the diameters of the disks such that at the steady-state distance ${r}_{bf}^{{\rm{ss}}}$, the area of the cells is constant, $A=0.29{R}_{{\rm{\max }}}^{2}$, regardless of the shape, i.e., the shape aspect ratio *σ*_*b*_/*σ*_*f*_. For the three shapes, the cell sizes are provided in Table 1. The length of the cells is then always of order *R*_max_.

### Cell noise

The dynamics of individual cells are often observed to fluctuate in time and space. To account for these random fluctuations in our model, we implemented cell noise. The noise, which is applied to all cell disks, is given by the force

11 $${\mathop{F}\limits^{\longrightarrow}}_{{\rm{noise}}}=\sqrt{2d}\,\mathop{\xi }\limits^{\longrightarrow}(t)$$

with both components of $\vec{\xi }(t)=({\xi }_{x}(t),{\xi }_{y}(t))$ being normally distributed random variables obeying 〈*ξ*_*i*_(*t*)〉 = 0 and 〈*ξ*_*i*_(*t*)*ξ*_*j*_(*t*′)〉 = *δ*_*ij*_ *δ*(*t* − *t*′) with Kronecker-*δ δ*_*ij*_ and *δ*-function *δ*(*t* − *t*′). As the simulation is performed with a finite time step Δ*t*, the force per timestep is

12 $${\mathop{F}\limits^{\longrightarrow}}_{{\rm{noise}}}=\sqrt{2d/{\rm{\Delta }}t}\,\mathop{\xi }\limits^{\longrightarrow}(t)$$

The timestep is 1.9 · 10^−4^ *τ*_mig_. The black line in Fig. 4(a) was calculated for *d* = 8.0 · 10^−4^ *R*_max_ *v* ^ss^.

### Derivation of the model from a model with a periodic crawling cycle

**Figure 12:**
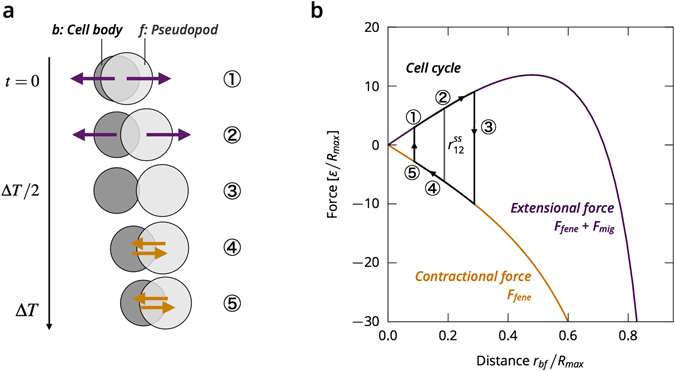
A multi-step cell migration cycle. (**a**) Illustration of the stages of the cell migration cycle. (**b**) The forces acting in a single cell on the two disks at a distance *r*_*bf*_. The cell migration cycle of (**a**) is marked as a black path. In the limit of the vanishing cycle period, the cell has a constant extension ${r}_{bf}^{{\rm{ss}}}$ where the forces exactly balance (marked by the grey line).

The cell migration mechanism presented in this letter can be derived from a coarse-graining of a two-stage periodic crawling cycle that is repeated with a time period Δ*T* (Fig. 12).

In the first stage of the crawling cycle 0 < *t* < Δ*T*/2, the pseudopod is pushed forward against friction with the substrate by an extensional force, *F*_fene_ + *F*_mig_, which acts between the two disks of opposite signs, while the cell body adheres to the substrate with *ζ*_*b*_ = ∞,

13 $${\mathop{v}\limits^{\longrightarrow}}_{b}(t)=\mathrm{0,}\quad {\mathop{v}\limits^{\longrightarrow}}_{f}(t)=\frac{{\mathop{F}\limits^{\longrightarrow}}_{{\rm{fene}}}+{\mathop{F}\limits^{\longrightarrow}}_{{\rm{mig}}}}{{\zeta }_{f}}.$$

In the second stage of the crawling cycle, Δ*T*/2 < *t* < Δ*T*, the pseudopod adheres to the substrate with *ζ*_*f*_ = ∞ and the cell body is drawn in with a contraction force ${\overrightarrow{F}}_{{\rm{fene}}}$,

14 $${\mathop{v}\limits^{\longrightarrow}}_{b}(t)=\frac{-{\mathop{F}\limits^{\longrightarrow}}_{{\rm{fene}}}}{{\zeta }_{b}},\quad {\mathop{v}\limits^{\longrightarrow}}_{f}(t)=0.$$

The velocities of the disks, averaged over the full cycle Δ*T* are then given by

15 $${\bar{\vec{v}}}_{f}=\frac{1}{{\zeta }_{f}{\rm{\Delta }}T}{\int }_{0}^{{\rm{\Delta }}T\mathrm{/2}}[{\mathop{F}\limits^{\longrightarrow}}_{{\rm{fene}}}({\mathop{r}\limits^{\longrightarrow}}_{bf}(t))+{F}_{{\rm{mig}}}({\mathop{r}\limits^{\longrightarrow}}_{bf}(t))]dt,$$

16 $${\bar{\vec{v}}}_{b}=-\frac{1}{{\zeta }_{b}{\rm{\Delta }}T}{\int }_{{\rm{\Delta }}T\mathrm{/2}}^{{\rm{\Delta }}T}{\mathop{F}\limits^{\longrightarrow}}_{{\rm{fene}}}({\mathop{r}\limits^{\longrightarrow}}_{bf}(t))dt.$$

In the limit Δ*T* → 0, ${\mathop{r}\limits^{\longrightarrow}}_{bf}(t)$ becomes a constant; thus,

17 $${\mathop{v}\limits^{\longrightarrow}}_{f}(t)=\frac{1}{2{\zeta }_{f}}[{\vec{F}}_{{\rm{fene}}}({\mathop{r}\limits^{\longrightarrow}}_{bf}(t))+{F}_{{\rm{mig}}}({\mathop{r}\limits^{\longrightarrow}}_{bf}(t))],$$

18 $${\mathop{v}\limits^{\longrightarrow}}_{b}(t)=-\frac{1}{2{\zeta }_{b}}{\mathop{F}\limits^{\longrightarrow}}_{{\rm{fene}}}({\mathop{r}\limits^{\longrightarrow}}_{bf}(t\mathrm{))}.$$

This limit can be interpreted as making the assumption that in a real cell, all the events of the cycle, namely, cytoskeleton contraction, formation of protrusions, adhesion to the substrate, and detachment from the substrate, occur simultaneously or in close succession. Eq. 5 is finally obtained by defining *ζ* = 2*ζ*_*b*_ = 2*ζ*_*f*_ and adding the cell-cell interaction force *F*_WCA_.

## Electronic supplementary material

Video to fig. 2a

Video to fig. 2b

Video to fig. 2c

Video to fig. 2d

Video to fig. 2e

Video to fig. 2f

Video to fig. 10a

Video to fig. 10b

Video to fig. 10c

Video to fig. 11a

Video to fig. 11b

Video to fig. 11c

Video showing cluster formation
